# Supplementary figures and images for: The alpha/B.1.1.7 SARS-CoV-2 variant exhibits significantly higher affinity for ACE-2 and requires lower inoculation doses to cause disease in K18-hACE2 mice
Source: eLife. 2021 Nov 25;10:e70002. doi: 10.7554/eLife.70002 (PMC8635972; doi:10.7554/eLife.70002)

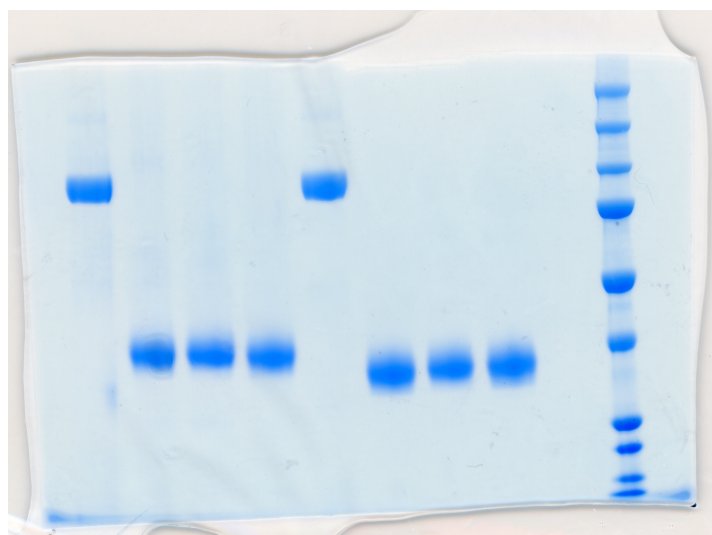

Supplement: Figure 1—source data 1. [file elife-70002-fig1-data1.pdf]

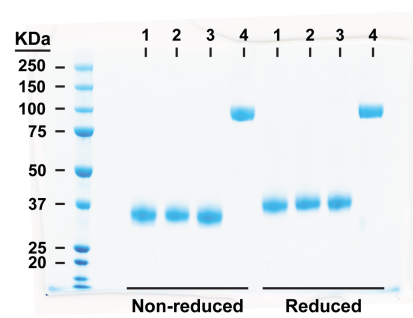

1. RBD wt
2. RBD N439K
3. RBD N501Y
4. ACE-2

Supplement: Figure 1—source data 2. [file elife-70002-fig1-data2.pdf]
